# Physician estimate of inflammation *vs* global assessment in explaining variations in swollen joint counts in rheumatoid arthritis patients

**DOI:** 10.1093/rap/rkae057

**Published:** 2024-04-27

**Authors:** Juan Schmukler, Tengfei Li, Theodore Pincus

**Affiliations:** Division of Rheumatology, Department of Medicine, Rush University School of Medicine, Chicago, IL, USA; Division of Rheumatology, Department of Medicine, Rush University School of Medicine, Chicago, IL, USA; Division of Rheumatology, Department of Medicine, Rush University School of Medicine, Chicago, IL, USA

**Keywords:** rheumatoid arthritis, disease activity, outcomes research

## Abstract

**Objective:**

To analyse patients with RA for inflammatory activity by physician estimate of global assessment (DOCGL) *vs* an estimate of inflammatory activity (DOCINF) to explain variation in the swollen joint count (SJC).

**Methods:**

Patients with RA were studied at routine care visits. Patients completed a multidimensional health assessment questionnaire (MDHAQ) and the physician completed a 28-joint count for swollen (SJC), tender (TJC) and deformed (DJC) joints and a RheuMetric checklist with a 0–10 DOCGL visual numeric scale (VNS) and 0–10 VNS estimates of inflammation (DOCINF), damage (DOCDAM) and patient distress (DOCSTR). The disease activity score in 28 joints with ESR (DAS28-ESR), Clinical Disease Activity Index (CDAI) and Routine Assessment of Patient Index Data 3 (RAPID3) were calculated. Individual scores and RA indices were compared according to Spearman correlation coefficients and regression analyses.

**Results:**

A total of 104 unselected patients were included, with a median age and disease duration of 54.5 and 5 years, respectively. The median DAS28-ESR was 2.9 (Q1–Q3: 2.0–3.7), indicating low activity. DOCINF was correlated significantly with DOCGL (ρ = 0.775). Both DOCGL and DOCINF were correlated significantly with most other measures; correlations with DOCGL were generally higher than with DOCINF other than for SJC. In regression analyses, DOCINF was more explanatory of variation in SJC than DOCGL and other DAS28-ESR components.

**Conclusions:**

Variation in SJC is explained more by a 0–10 DOCINF VNS than the traditional DOCGL or any other measure in RA patients seen in routine care. DOCINF on a RheuMetric checklist can provide informative quantitative scores concerning inflammatory activity in RA patients monitored over long periods.

Key messages

- A physician estimate of inflammation predicted variation in the SJC to a greater extent than the traditional physician estimate of global assessment or other measures in RA patients seen in routine care.
- Incorporation of a RheuMetric physician checklist would allow for quantitative documentation of multiple domains of disease status in RA, i.e. inflammatory activity, irreversible damage and patient distress.

## Introduction

The core dataset of seven measures to assess patients with RA [1, 2] were identified initially for use in clinical trials, in which each functions effectively to recognize group differences between active and control treatments [3–8]. However, all six clinical core dataset measures—swollen joint count (SJC), tender joint count (TJC), physician global assessment (DOCGL), patient physical function (FN), pain and patient global assessment (PATGL) (other than ESR or CRP) [1, 2]—may be elevated in patients with RA with comorbid FM [9, 10], depression [11–13], osteoarthritis [14, 15] and other comorbidities, even in a setting of little to no inflammatory activity. These elevations may affect RA indices such as the disease activity score in 28 joints (DAS28) [16, 17] and Clinical Disease Activity Index (CDAI) [18], complicating interpretation to recognize improvement [19], treat-to-target [20] and general management.

The RA core dataset measures that are least likely to be elevated in patients with comorbid FM are ESR (or CRP) and SJC [21], while TJC appears most likely to be elevated. Often the SJC is not available, as most rheumatologists do not perform a formal joint count in most patients with RA [22] unless required for the patient to gain access to a therapy. Therefore, the only quantitative measures found in medical records of most patients with RA are ESR and/or CRP, which are normal in 45% of patients at presentation [23] and unchanged in some patients whose clinical signs of inflammatory activity are resolved [24]. Documentation of improved patient status according to narrative descriptions by the physician rather than quantitative data from both the patient and physician limits recognition of the value of rheumatology care for the individual patient, medical community and reimbursement agencies.

These considerations suggest that a pragmatic and feasible surrogate for SJC to assess inflammatory activity quantitatively in busy clinical settings would appear to be an unmet need for routine patient care. One possible candidate may be a 0–10 DOCGL, but this measure, as with all clinical core dataset measures, is recognized to be elevated in patients with non-inflammatory comorbidities, which are variably incorporated into the DOCGL by rheumatologists in routine care [25]. A possibly more specific and pragmatic measure is a 0–10 physician visual numeric scale (VNS) estimate for inflammation (DOCINF) on a physician RheuMetric checklist, accompanied by 0–10 VNSs for joint damage (DOCDAM) and patient distress (DOCSTR). Face validity and content validity of these RheuMetric checklist subglobal estimates have been reported [26, 27]. In this report, we analyse whether DOCINF may explain variations in SJC, which appears an optimal indicator of inflammatory activity that is as effective or more effective than DOCGL.

## Methods

### Patients

Non-selected routine care patients who met the 2010 ACR/EULAR classification criteria for RA seen at one community rheumatology setting between February and April 2022 were studied.

### Institutional approval

All information was collected as part of routine clinical care, exempt from informed consent and institutional review board approval, and entered into a de-identified database.

### Patient-reported measures

All patients seen at the study site, regardless of diagnosis, are asked to complete a multidimensional health assessment questionnaire (MDHAQ) [28, 29] as part of the clinical encounter. The MDHAQ is a two-page, single-sheet questionnaire developed from the Stanford HAQ [30]. The MDHAQ queries 10 patient self-report scores for physical function (FN) on a 0–3 scale, as in the HAQ, for a total score of 0–30, which is divided by 3 for a 0–10 score. Pain, patient global assessment (PATGL) and fatigue are assessed on a scale of 0–10 VNS in increments of 0.5 units. Three psychological items for sleep quality, anxiety and depression are queried in the patient-friendly HAQ format for a total of 0–9.9 as a psychological scale. A self-report painful joint count, modified from an RA Disease Activity Index (RADAI), adds neck and back to the 16 joints in the original RADAI for 18 joints scored 0–3, giving a total score of 0–54. A 60-symptom checklist queries yes/no responses to common symptoms, including depression, and can be used as a review of systems and to recognize adverse events to medications.

Four indices may be calculated from MDHAQ scores without an additional questionnaire: Routine Assessment of Patient Index Data 3 (RAPID3) [31] to assess clinical status, which has been found informative to assess patient status in all rheumatic diseases studied [32–34]; Fibromyalgia Assessment Screening Tool (FAST3F) [35, 36], which is a composite index to screen for FM derived from three MDHAQ scales, in which 1 point each is awarded for a fatigue score >6, a 60-symptom checklist score ≥16 and a self-reported painful joint count RADAI ≥16. A FAST3F score of 2–3 agrees >80% with the formal 2011 revised FM criteria [35]. The MDHAQ anxiety screen (MAS2) [37] and MDHAQ depression screen (MDS2) [38] indices agree 80% with reference questionnaires for anxiety and depression, respectively.

### Physician measures

**Figure 1.**
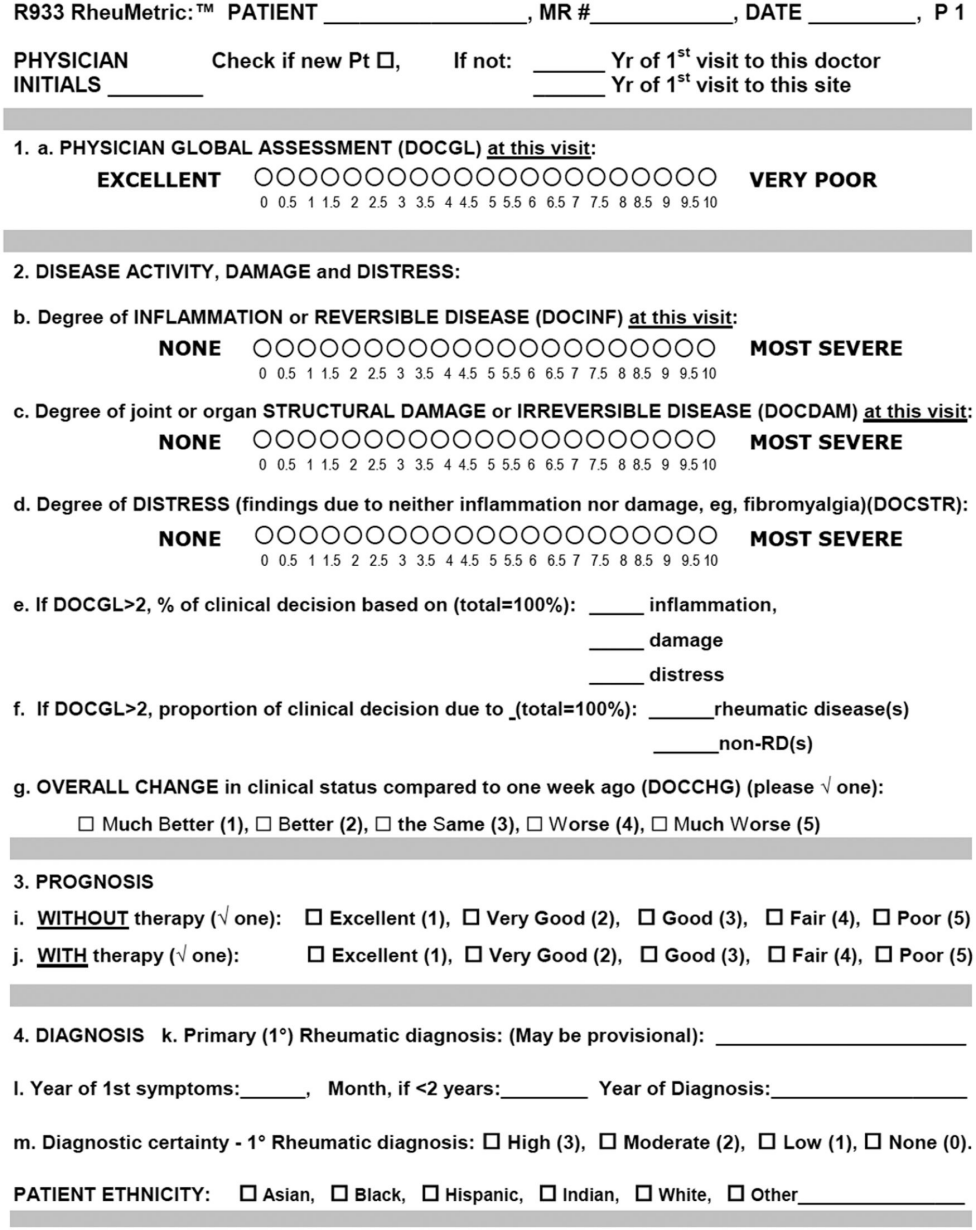
RheuMetric physician checklist

A single assessor (J.S.) performed a formal 28-joint count [39] that included SJC, TJC and deformed joint count (DJC) in all patients. This assessor also completed a RheuMetric checklist [40] (Fig. 1) to score a 0–10 VNS for DOCGL and three 0–10 VNSs for DOCINF, DOCDAM and DOCNON.

### RA indices

RAPID3 scores were obtained from MDHAQ responses. The DAS28 with ESR (DAS28-ESR) was calculated based on SJC, TJC, PATGL (from the MDHAQ) and ESR [16, 17] (if ESR was available as part of routine care). The CDAI scores were calculated based on SJC, TJC, PATGL [18] (from the MDHAQ) and DOCGL from the RheuMetric checklist.

### Statistical analyses

MDHAQ, joint count and RheuMetric data were entered into a de-identified database. Mean (s.d.) and median [interquartiles (Q1, Q3)] were presented for continuous demographic variables and count with percentage for categorical demographic variables, including MDHAQ individual scores and indices; 28-joint counts for SJC, TJC and DJC; and RheuMetric scores for DOCGL, DOCINF, DOCDAM and DOCSTR. Spearman correlation coefficients of individual MDHAQ scores and indices and DOCGL, DOCINF, DOCDAM and DOCSTR were calculated with significance tests. Multiple linear regression analyses were performed with each RheuMetric scale and SJC as the dependent variables, respectively, with several candidate independent variables chosen based on biologic plausibility and prior reports.

## Results

### Study patients

The study included 104 RA patients. The mean age was 53.1 years (s.d. 14.3) and 83% were female (Table 1). The mean disease duration was 6.7 years (s.d. 7.2). The mean SJC, TJC and DJC were 1.4 (s.d. 3.0), 2.3 (s.d. 4.5) and 2.0 (s.d. 4.1), respectively (Table 1).

**Table 1.** Demographic and clinical characteristics and index scores of 104 patients with RA

Characteristics	Median (Q1, Q3) or n (%)	Mean (S.D.)
Female, *n* (%)	86 (83%)	
Age, years, median	54.5 (41.5, 64.0)	53.1 (14.3)
Duration of disease, years, median	5 (1.4, 9.0)	6.7 (7.2)
RAPID3, median	10.8 (4.8, 18.8)	12.2 (8.6)
Physical function (0–10), median	2.0 (0.5, 4.8)	3.0 (3.1)
Pain (0–10), median	5.0 (2.0, 7.5)	4.8 (3.1)
PATGL (0–10), median	4.0 (1.5, 7.0)	4.4 (3.2)
28-joint SJC	0 (0, 1.5)	1.4 (3.0)
28-joint TJC	0 (0, 2.5)	2.3 (4.5)
28-joint DJC	0 (0, 2.0)	2.0 (4.1)
ESR, mm/h, median	22.5 (12.0, 39.0)	27.6 (20.3)
CDAI, median	10.5 (4.0, 15.8)	12.0 (10.3)
DAS28-ESR^a^, median	2.9 (2.0, 3.7)	2.9 (1.3)
DOCGL (0–10), median	4.0 (2.0, 5.5)	3.8 (2.3)
DOCINF (0–10), median	2.0 (0.5, 3.5)	2.5 (2.2)
DOCDAM (0–10), median	2.0 (0.5, 4.0)	2.6 (2.3)
DOCSTR (0–10), median	1.5 (0.8, 3.0)	2.1 (1.8)
Positive anxiety screen on MDHAQ, *n* (%)	22 (26)	
Positive depression screen on MDHAQ, *n* (%)	17 (20)	
Positive FM screen on MDHAQ, *n* (%)	17 (16)	

a DAS28-ESR available for 70/104 patients.

b MDS2 available for 86/104 patients.

The median DAS28-ESR was 2.9 (Q1–Q3: 2.0–3.7), indicating low activity; CDAI was 10.5 (Q1–Q3: 4.0–15.8), indicating moderate activity; and RAPID3 was 10.8 (Q1–Q3: 4.8–18.8), indicating moderate severity (RAPID3 levels have been defined according to ‘severity’ rather than ‘activity’ [8]) (Table 1). Among the 104 patients, 26% screened positive for anxiety, 20% for depression and 16% for FM (Table 1) according to MDHAQ indices.

### Correlations of DOCGL and subglobal estimates with other measures

Correlations greater than *r* = 0.6 of DOCGL were seen with DOCINF, DOCDAM, TJC, physical function, pain, PATGL, RADAI self-report painful joint count, DAS28-CRP, DAS28-ESR, CDAI (of which it is a component), RAPID3 and FAST3F (Table 2). Correlations greater than *r* = 0.6 of DOCINF were seen with DOCGL, SJC, TJC, pain, DAS28-ESR, DAS28-CRP and CDAI. Correlations greater than *r* = 0.6 of DOCDAM were seen with DOCGL and DJC. Correlations greater than *r* = 0.6 of DOCSTR were seen with pain VNS and PATGL. Correlations greater than *r* = 0.6 of PATGL were seen with DOCGL, DOCSTR, physical function, pain, fatigue, RADAI self-report painful joint count, DAS28-CRP (of which it is a component), CDAI (of which it is a component), RAPID3 (of which it is a component) and FAST3F (Table 2).

**Table 2.** Correlation matrix for DOCGL, DOCINF, DOCDAM and DOCSTR with various candidate variables

Variables	DOCGL	DOCINF	DOCDAM	DOCSTR	PATGL
Age	0.114	−0.126	**0.500** **	0.178	0.125
Duration	**0.197** *	−0.094	**0.453** **	**0.217** *	0.154
DOCGL	1	**0.775** **	**0.600** **	**0.582** **	**0.846** **
DOCINF	**0.75** **	1	0.224	0.291	**0.587** **
DOCDAM	**0.600** **	0.224	1	**0.447** *	**0.509** **
DOCSTR	**0.582** **	0.291	**0.447** *	1	**0.674** **
SJC	**0.525** *	**0.613** **	0.165	0.090	**0.362** *
TJC	**0.673** **	**0.637** **	0.305	0.313	**0.552** *
DJC	**0.393** **	0.098	**0.698** **	0.144	0.270
ESR	**0.392** **	0.304*	**0.274** *	0.209	**0.204** *
CRP	**0.445** *	0.269	**0.379** *	0.184	0.348
FN	**0.661** **	**0.382** *	**0.501** **	**0.573** **	**0.709** **
Pain	**0.856** **	**0.603** **	**0.542** **	**0.657** **	**0.924** **
PATGL	**0.846** **	**0.587** **	**0.509** **	**0.674** **	1
Fatigue	**0.566** **	**0.374** *	**0.321** *	**0.549** **	**0.685** **
RADAI	**0.683** **	**0.536** **	**0.381** *	**0.540** **	**0.660** **
Symptoms	**0.470** **	**0.392** **	0.224	**0.423** **	**0.476** **
DAS28-CRP	**0.732** **	**0.696** **	**0.386** *	0.336	**0.609** **
DAS28-ESR	**0.673** **	**0.681** **	**0.323** *	**0.273** *	**0.51** **
CDAI	**0.925** **	**0.763** **	**0.494** **	**0.591** *	**0.876** **
RAPID3	**0.859** **	**0.569** **	**0.573** **	**0.689** **	**0.959** **
FAST3F	**0.646** **	**0.476** **	**0.277** *	**0.531** **	**0.645** **
MDS2	**0.315** *	0.137	0.233	**0.208** *	**0.370** *
MAS	0.257	0.172	0.100	0.234	**0.364** *

Data are Spearman correlations.

Bold text indicates statistically significant values.

* *P* < 0.05,

** *P* < 0.001.

Many other statistically significant correlations were seen between DOCGL, DOCINF, DOCDAM, DOCSTR and PATGL with other variables on the MDHAQ (Table 2), but these appear to be of lesser clinical significance. DOCINF was correlated at low levels (*r* < 0.4) with DOCDAM and DOCSTR, and DOCDAM was correlated with DOCSTR at lower levels than seen for correlations with other measures and indices (Table 2). These findings indicate specificity for the three 0–10 DOCINF, DOCDAM and DOCSTR estimates, although all were correlated at high levels (*r* > 0.58) with DOCGL.

### Regressions to analyse variations of DOCINF, DOCDAM and DOCSTR according to other variables

Three multiple linear regressions were performed with each RheuMetric 0–10 subscale (DOCINF, DOCDAM and DOCSTR) as dependent variables and variables correlated significantly with the dependent variable as independent variables (Table 3). Since including both pain and PATGL would cause issues of multicollinearity, we included pain instead of PATGL in Table 3 and PATGL instead of pain and both pain and PATGL in Supplementary Table S1, available at *Rheumatology Advances in Practice* online. DOCINF was explained significantly by SJC, TJC and pain, indicating that 54% of the variation in DOCINF was explained by model 1. Variation in DOCDAM was explained by DJC and pain as the significant variables, indicating that 61% of the variation in DOCDAM was explained by model 2. Variation in DOCSTR was explained significantly by pain and fatigue, with 39% of the variation in DOCSTR being explained by model 3.

**Table 3.** Multiple linear regression models with physician estimates of inflammatory activity (DOCGL, model 1), damage (DOCDAM, model 2) and patient distress (DOCSTR, model 3) as dependent variables, with multiple candidate explanatory variables

Variables	Model 1: DOCINF, β	Model 2: DOCDAM, β	Model 3: DOCSTR, β
	(95% CI)	(95% CI)	(95% CI)
SJC	**0.21** **(0.08, 0.34)** *		
TJC	0.09 (0.01, 0.18)*		
DJC		**0.27** **(0.19, 0.35)** **	
FN	−0.03 (−0.15, 0.10)	0.08 (−0.04, 0.20)	
Pain	**0.34** **(0.19, 0.48)** **	0.25 (0.12, 0.38)*	**0.20** (0.08, 0.32)*
Fatigue	0.02 (−0.11, 0.14)	−0.003 (−0.12, 0.12)	**0.15** **(0.005, 0.29)** *
Age	−0.02 (−0.04, 0.004)	0.02 (−0.004, 0.04)	0.01 (−0.01, 0.04)
Disease duration	−0.03 (−0.08, 0.01)	0.03 (−0.02, 0.07)	0.02 (−0.03, 0.06)
Male sex	0.31 (−0.48, 1.10)		−0.60 (−1.51, 0.31)
FAST3F			0.73 (−0.28, 1.74)
MDS2			−0.18 (−1.16, 0.80)
MAS			−0.10 (−0.99, 0.78)
Adjusted *R*^2^	0.54	0.61	0.39

Bold text indicates statistically significant values.

* *P* < 0.05,

** *P* < 0.0001.

### Regressions to analyse variations of SJC according to other variables

Further regressions were performed with SJC as the dependent variable and different measures as potential independent variables (Table 4). Model 4 included only DOCGL and DOCINF; DOCINF was more explanatory than DOCGL for variations in SJC. Model 5 added the two other RheuMetric physician subglobal estimates, DOCDAM and DOCSTR. DOCINF was again the most explanatory variable of variation in SJC, indicating the specificity of DOCINF (Table 4). Model 6 included DOCINF and the three DAS28 components beyond SJC, TJC, PATGL and ESR. Again, DOCINF was the most explanatory variable and the only significantly explanatory variable for variations in SJC. Model 7 added DOCGL to model 6 and deleted ESR. In this model, both DOCINF and TJC were significantly explanatory of variations in SJC. In all the models, DOCINF was more explanatory than DOCGL for variations in SJC (Table 4).

**Table 4.** Multiple linear regression models with SJC as the dependent variable and different candidate explanatory variables

Variables	Model 4, β	Model 5, β	Model 6, β	Model 7, β
	(95% CI)	(95% CI)	(95% CI)	(95% CI)
DOCGL	0.02 (−0.32, 0.35)	−0.01 (−0.59, 0.57)		0.07 (−0.39, 0.53)
DOCINF	**0.74** ** (**0.39, 1.08)**	**0.76** * (**0.30, 1.21)**	**0.67** * (**0.27, 1.08)**	**0.46** * (**0.12, 0.80)**
DOCDAM		0.18 (−0.14, 0.49)		
DOCSTR		−0.23 (−0.59, 0.12)		
TJC			0.12 (−0.07, 0.31)	**0.28** ** (**0.16, 0.40)**
PATGL			−0.11 (−0.34, 0.11)	−0.10 (−0.35, 0.16)
ESR			0.03 (−0.01, 0.06)	
Adjusted *R*^2^	0.30	0.31	0.36	0.42

Bold text indicates statistically significant values.

* *P* < 0.05,

** *P* < 0.001.

## Discussion

This report indicates that DOCINF, a global estimate of inflammatory activity, is more explanatory of variations in SJC than DOCGL in RA patients seen in routine care. Although DOCGL is more likely to distinguish active from control treatments than any of the other six core dataset measures in clinical trials [3, 5, 6], DOCGL (as all clinical RA core dataset measures) is likely to be affected by comorbidities, particularly in routine clinical care. Different clinicians might vary in their approach to scoring DOCGL [25]—some might be based only on inflammatory activity while others might consider other patient problems beyond inflammation, such as joint damage [14], FM [9] and depression [11]. Therefore, individual subglobal assessments for inflammatory activity (DOCINF), joint damage (DOCDAM) and patient distress (DOCSTR) on a RheuMetric checklist may be more informative than DOCGL for clinical decisions concerning patient management [26, 27].

SJC, ESR and CRP appear least likely to be elevated by FM and other comorbidities, and therefore are the most specific clinical measures to indicate inflammatory activity [21]. Yet ESR and CRP are normal at presentation in at least 40% of patients with RA [23], are not decreased in some patients who show substantial clinical improvement [41] and are less likely than SJC or patient self-report measures to distinguish active from control treatment in clinical trials [3]. Therefore, SJC may be regarded as an optimal representation of inflammatory activity [42].

It should be emphasized that DOCINF requires a careful joint examination to assess swollen and tender joints, as well as joints with deformity/limited motion (as in the initial report of the 28 joint count [39]). A report concerning a mean of 94 s to score a 28-joint count, 114 s for DAS28 and 106 s for CDAI [43] did not distinguish between the time to perform a careful joint examination *vs* the time to record a formal joint count or indices. Preliminary data indicate that only 15–30 s are required to perform a careful 28-joint examination and an additional 30 s to record a RheuMetric checklist, for a total of 30–60 s (Schmukler and Pincus, manuscript in preparation). Therefore, much of the 94 s reported to score a 28-joint count involved recording the findings rather than performing the joint examination.

Another approach to analyse the time required to record different measures is to recognize that a 28-joint TJC and SJC requires 54 measures (swelling of shoulders is not recorded), compared with 16 measures on a RheuMetric checklist for DOCGL, DOCINF, DOCDAM, DOCSTR, the proportion of DOCGL attributed to inflammation, damage and distress, and additional measures. Recording of 54 *vs* 16 (3.3 times as many) measures requires more time for entry into a paper format or clicks in a computer format. It may be possible to record a paper format of a 28-joint count in a patient with 0 SJC, 0 TJC and 0 DJC, but that pertains to relatively few patients, as the median TJC was 2 and median DJC was 4.

As noted, most rheumatologists do not perform formal joint counts in busy routine clinical care settings [22] unless required for access of a specific individual patient to a therapy. It should be emphasized that DOCINF requires a careful joint examination to assess swollen and tender joints, as well as joints with deformity/limited motion. It matters considerably whether an RA patient has, say, 1, 11 or 21 swollen joints or 2, 12 or 22 swollen joints, but generally not whether the patient has 1 or 2, 11 or 12, or 21 or 22 swollen joints, which is within the error of the method [44–46]. The time saved in not recording a formal joint count might allow more doctor–patient communication concerning medications, adverse events, prognosis and other matters regarded as important by the patient and/or physician.

Several limitations are seen in this study. Only 104 patients at a single centre were included, although results are similar to a previous report from another setting [27]. The patients had a median disease duration of 6.7 years, with a median SJC of 0 and a mean of 1.42, indicating an overall low level of active inflammation. These results reflect more effective control of inflammatory activity than in previous decades and may even underestimate DOCINF as a surrogate for SJC. The study is cross-sectional, and longitudinal comparisons of DOCGL, DOCINF, DOCDAM and DOCSTR to monitor patients with RA over long periods would be of value.

In conclusion, variations in SJC are explained more by a 0–10 DOCINF VNS than the traditional DOCGL in patients with RA monitored in routine clinical care. The findings suggest that recording of DOCINF, DOCDAM and DOCSTR, in addition to DOCGL, on a RheuMetric checklist could provide informative quantitative scores concerning the status of patients with RA monitored over long periods. This approach would appear especially valuable when formal quantitative joint counts are not performed and/or when joint counts do not record DJC. DOCINF appears more informative to explain variations in SJC than DOCGL.

## Supplementary Material

rkae057_Supplementary_Data

## Data Availability

The dataset generated from this study is available upon request to the authors.
