# Supplementary material for: Physician estimate of inflammation vs global assessment in explaining variations in swollen joint counts in rheumatoid arthritis patients
Source: Rheumatol Adv Pract. 2024 Apr 27;8(2):rkae057. doi: 10.1093/rap/rkae057 (PMC11116827; doi:10.1093/rap/rkae057)
Supplement: rkae057_Supplementary_Data [file rkae057_supplementary_data.docx]

**Supplementary Table S1:** Multiple linear regression models with physician estimates of inflammatory activity (DOCGL, models 1 and 1´), damage (DOCDAM, models 2 and 2´) and patient distress (DOCSTR, models 3 and 3´) as dependent variable, with multiple candidate explanatory variables.

|  | Model 1’:  DOCINF  B (95% CI) | Model 1’’:  DOCINF  B (95% CI) | Model 2’:  DOCDAM  B (95% CI) | Model 2’’:  DOCDAM  B (95% CI) | Model 3’:  DOCSTR  B (95% CI) | Model 3’’:  DOCSTTR  B (95% CI) |
| --- | --- | --- | --- | --- | --- | --- |
| SJC | 0.21  (0.09, 0.34)* | 0.21  (0.08, 0.34)* |  |  |  |  |
| TJC | **0.10**  **(0.02, 0.19)*** | **0.09**  **(0.01 , 0.18)*** |  |  |  |  |
| DJC |  |  | **0.27**  **(0.20, 0.35**)** | **0.27**  **(0.19, 0.35**)** |  |  |
| FN | -0.05  (-0.19, 0.08) | -0.04  (-0.18 , 0.09) | 0.06  (-0.06, 0.19) | 0.07  (-0.06, 0.20) |  |  |
| Pain |  | 0.24  (-0.03 , 0.51) |  | 0.20  (-0.06, 0.45) |  | 0.06  (-0.23, 0.34) |
| PATGL | 0.33  (0.18, 0.49)** | 0.12  (-0.16 , 0.40) | 0.24  (0.10, 0.39)* | 0.06  (-0.21, 0.33) | 0.22  (0.09, 0.35)* | 0.17  (-0.14, 0.47) |
| Fatigue | 0.0003  (-0.13, 0.13) | 0.002  (-0.13 , 0.13) | -0.01  (-0.14, 0.11) | -0.01  (-0.14, 0.11) | 0.12  (-0.03, 0.26) | 0.12  (-0.02, 0.27) |
| Age | -0.02  (-0.04, 0.01) | -0.02  (-0.04, 0.005) | 0.02  (-0.003, 0.04) | 0.02  (-0.004, 0.04) | 0.01  (-0.01, 0.03) | 0.01  (-0.01, 0.03) |
| Disease duration | -0.03  (-0.07, 0.02) | -0.03  (-0.07, 0.02) | 0.03  (-0.01, 0.08) | 0.03  (-0.02, 0.07) | 0.02  (-0.02, 0.07) | 0.02  (-0.02, 0.07) |
| Male sex | 0.16  (-0.63, 0.95) | 0.28  (-0.52, 1.07) |  |  | -0.59  (-1.49, 0.32) | -0.58  (-1.49, 0.32) |
| FAST3F |  |  |  |  | 0.73  (-0.27, 1.73) | 0.72  (-0.30, 1.73) |
| MDS2 |  |  |  |  | -0.19  (-1.17, 0.78) | -0.19  (-1.17, 0.79) |
| MAS |  |  |  |  | -0.13  (-1.01, 0.75) | -0.12  (-1.00, 0.77) |
| Adjusted R^2^ | 0.53 | 0.54 | 0.62 | 0.60 | 0.40 | 0.39 |

Data are Beta coefficients and 95% confidence intervals

SJC= swollen joint count, TJC= tender joint count, DJC= deformed joint count, FN= physical function scale on MDHAQ, PATGL= patient global assessment, FAST3F= fibromyalgia assessment screening tool, MDS2= MDHAQ depression screening, MAS2= MDHAQ anxiety screening

*p <0.05

** p< 0.0001

Bold text indicates statistically significant values.
